# 3-Nitro-*N*-[(pyrrolidin-1-yl)carbothioyl]benzamide

**DOI:** 10.1107/S1600536812021319

**Published:** 2012-05-19

**Authors:** Nursakinah Zulkifli, Siti Fairus M. Yusoff, Bohari M. Yamin

**Affiliations:** a School of Chemical Sciences and Food Technology, Universiti Kebangsaan Malaysia, 43600 Bangi, Selangor, Malaysia

## Abstract

In the mol­ecule of the title compound, C_12_H_13_N_3_O_3_S, the pyrrolidine ring adopts a half-chair conformation and the dihedral angle formed by the nitro group with the benzene ring is 15.18 (18)°. In the crystal, mol­ecules are linked by N—H⋯S and C—H⋯O inter­molecular hydrogen bonds into chains parallel to the *c* axis.

## Related literature

For standard bond-length data, see: Allen *et al.* (1987 ▶). For related structures, see: Emen *et al.* (2003 ▶); Kayhan *et al.* (2003 ▶).
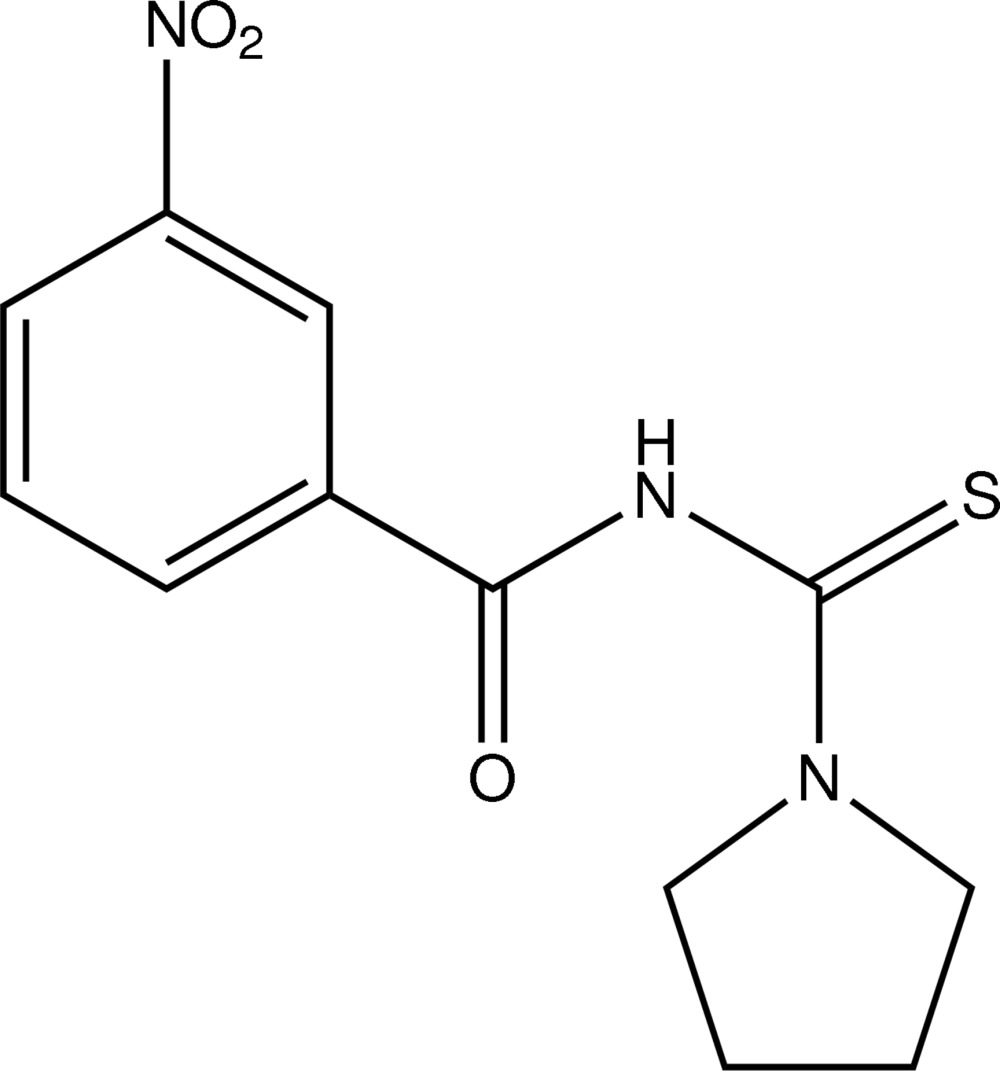


## Experimental

### 

#### Crystal data

C_12_H_13_N_3_O_3_S
*M* *_r_* = 279.31
Monoclinic, 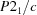

*a* = 11.331 (3) Å
*b* = 13.543 (3) Å
*c* = 8.5982 (19) Å
β = 97.168 (5)°
*V* = 1309.2 (5) Å^3^
*Z* = 4
Mo *K*α radiation
μ = 0.26 mm^−1^
*T* = 298 K
0.28 × 0.22 × 0.09 mm

#### Data collection

Bruker SMART APEX CCD area-detector diffractometer
Absorption correction: multi-scan (*SADABS*; Sheldrick, 1996 ▶) *T*_min_ = 0.932, *T*_max_ = 0.977
7272 measured reflections
2288 independent reflections
1853 reflections with *I* > 2σ(*I*)
*R*_int_ = 0.031

#### Refinement

*R*[*F* ^2^ > 2σ(*F* ^2^)] = 0.055
*wR*(*F* ^2^) = 0.135
*S* = 1.08
2288 reflections
176 parameters
1 restraint
H atoms treated by a mixture of independent and constrained refinement
Δρ_max_ = 0.29 e Å^−3^
Δρ_min_ = −0.18 e Å^−3^

### 

Data collection: *SMART* (Siemens, 1996 ▶); cell refinement: *SAINT* (Siemens, 1996 ▶); data reduction: *SAINT*; program(s) used to solve structure: *SHELXS97* (Sheldrick, 2008 ▶); program(s) used to refine structure: *SHELXL97* (Sheldrick, 2008 ▶); molecular graphics: *SHELXTL* (Sheldrick, 2008 ▶); software used to prepare material for publication: *SHELXTL*, *PARST* (Nardelli, 1995 ▶) and *PLATON* (Spek, 2009 ▶).

## Supplementary Material

Crystal structure: contains datablock(s) global, I. DOI: 10.1107/S1600536812021319/rz2750sup1.cif

Structure factors: contains datablock(s) I. DOI: 10.1107/S1600536812021319/rz2750Isup2.hkl

Supplementary material file. DOI: 10.1107/S1600536812021319/rz2750Isup3.cml

Additional supplementary materials:  crystallographic information; 3D view; checkCIF report

## Figures and Tables

**Table 1:** Hydrogen-bond geometry (Å, °)

*D*—H⋯*A*	*D*—H	H⋯*A*	*D*⋯*A*	*D*—H⋯*A*
N2—H2⋯S1^i^	0.86 (2)	2.55 (2)	3.406 (3)	173
C1—H1⋯O3^ii^	0.93	2.48	3.169 (3)	131
C9—H9*A*⋯O3^ii^	0.97	2.32	3.274 (4)	167

Symmetry codes: (i) 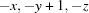; (ii) 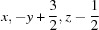.

**Fig. 1.**
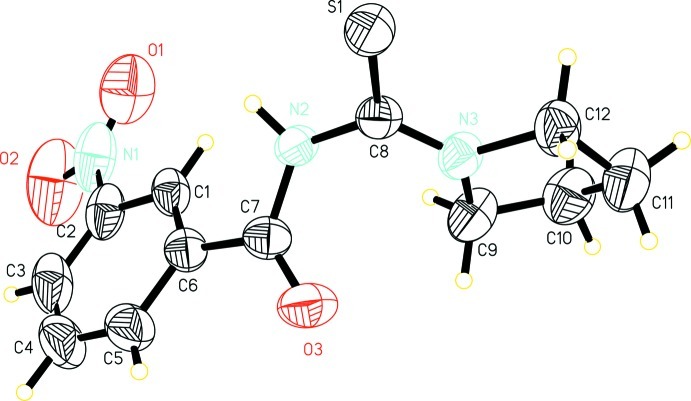
The molecular structure of the title compound with 50% probability displacement ellipsoids.

**Fig. 2.**
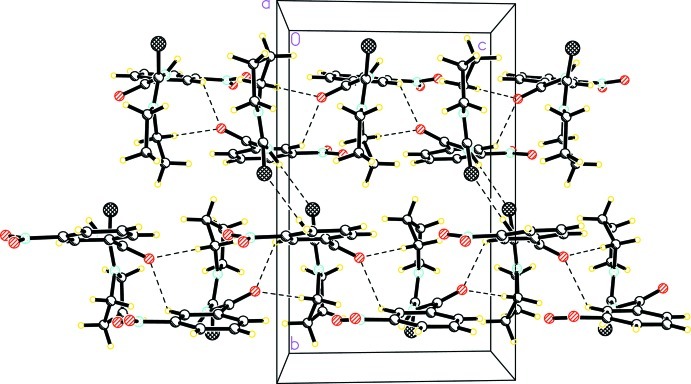
Packing diagram of the title compound, viewed down a axis. Dashed lines denote hydrogen bonds.

## supplementary crystallographic information

### Comment

The rapid increase in the number of syntheses of thiourea derivatives is driven by their applications in various fields including biology, pharmacy and materials for devices. The title compound is a thiourea derivative analogue to N'-(4-chlorobenzoyl)-N-(pyrrolidin-1-yl)thiourea (Kayhan *et al.*, 2003) and N'-(2-chlorobenzoyl)-N-(pyrrolidin-1-yl) (Emen *et al.*, 2003), except for the nitro substituent attached at 3-position of the benzene ring (Fig. 1). The molecule maintains a twisted conformation, but the C8-N2-C7-O3 torsion angle of 1.6 (4)° is a little larger than that found in N'-(2-chlorobenzoyl)-N-(pyrrolidin-1-yl)thiourea (0.42 (4)°). The C7-N2-C8-N3 torsion angle of 57.9 (4)° is comparable. The pyrrolidine ring adopts a half chair conformation. The bond lengths (Allen *et al.*, 1987) and angles are in normal ranges. In the crystal structure, the molecules are linked by N2–H2···S1, C1–H1···O3 and C9–H9A···O3 intermolecular hydrogen bonds (Table 1) to form chains parallel to the *c* axis (Fig. 2).

### Experimental

An ethanolic solution (10 ml) of 3-nitrobenzoyl isothiocyanate (0.416 g, 2 mmol) was added into a beaker containing pyrrolidine (0.007 g, 1 mmol) in 10 ml ethanol. The solution was refluxed for about 1 hour and left to evaporate at room temperature. Some colourless crsytals were obtained after 3 days on slow evaporation of the solvent. M. p.: 395.3-396.5 K.

### Refinement

The imino hydrogen atom was located in a difference Fourier map and refined isotropically with the N—H distance restrained to be 0.87 (1) Å. All other H-atoms were fixed geometrically at ideal positions and allowed to ride on the parent atoms, with C-H = 0.93-0.97Å, and with *U*_iso_(H) = 1.2 *U*_eq_(C).

### Crystal data

**Table:**

C_12_H_13_N_3_O_3_S	*F*(000) = 584
*M**_r_* = 279.31	*D*_x_ = 1.417 Mg m^−^^3^
Monoclinic, *P*2_1_/*c*	Mo *K*α radiation, λ = 0.71073 Å
Hall symbol: -P 2ybc	Cell parameters from 2210 reflections
*a* = 11.331 (3) Å	θ = 1.8–25.0°
*b* = 13.543 (3) Å	µ = 0.26 mm^−^^1^
*c* = 8.5982 (19) Å	*T* = 298 K
β = 97.168 (5)°	Slab, colourless
*V* = 1309.2 (5) Å^3^	0.28 × 0.22 × 0.09 mm
*Z* = 4	

### Data collection

**Table:**

Bruker SMART APEX CCD area-detector diffractometer	2288 independent reflections
Radiation source: fine-focus sealed tube	1853 reflections with *I* > 2σ(*I*)
Graphite monochromator	*R*_int_ = 0.031
Detector resolution: 83.66 pixels mm^-1^	θ_max_ = 25.0°, θ_min_ = 1.8°
ω scan	*h* = −12→13
Absorption correction: multi-scan (*SADABS*; Sheldrick, 1996)	*k* = −15→16
*T*_min_ = 0.932, *T*_max_ = 0.977	*l* = −10→10
7272 measured reflections	

### Refinement

**Table:**

Refinement on *F*^2^	Primary atom site location: structure-invariant direct methods
Least-squares matrix: full	Secondary atom site location: difference Fourier map
*R*[*F*^2^ > 2σ(*F*^2^)] = 0.055	Hydrogen site location: inferred from neighbouring sites
*wR*(*F*^2^) = 0.135	H atoms treated by a mixture of independent and constrained refinement
*S* = 1.08	*w* = 1/[σ^2^(*F*_o_^2^) + (0.063*P*)^2^ + 0.5394*P*] where *P* = (*F*_o_^2^ + 2*F*_c_^2^)/3
2288 reflections	(Δ/σ)_max_ = 0.001
176 parameters	Δρ_max_ = 0.29 e Å^−^^3^
1 restraint	Δρ_min_ = −0.18 e Å^−^^3^

### Special details

**Table:**

Geometry. All esds (except the esd in the dihedral angle between two l.s. planes) are estimated using the full covariance matrix. The cell esds are taken into account individually in the estimation of esds in distances, angles and torsion angles; correlations between esds in cell parameters are only used when they are defined by crystal symmetry. An approximate (isotropic) treatment of cell esds is used for estimating esds involving l.s. planes.
Refinement. Refinement of F^2^ against ALL reflections. The weighted R-factor wR and goodness of fit S are based on F^2^, conventional R-factors R are based on F, with F set to zero for negative F^2^. The threshold expression of F^2^ > 2sigma(F^2^) is used only for calculating R-factors(gt) etc. and is not relevant to the choice of reflections for refinement. R-factors based on F^2^ are statistically about twice as large as those based on F, and R- factors based on ALL data will be even larger.

### Fractional atomic coordinates and isotropic or equivalent isotropic displacement parameters (Å^2^)

**Table:**

	*x*	*y*	*z*	*U*_iso_*/*U*_eq_	
S1	−0.14867 (6)	0.55605 (5)	0.11047 (10)	0.0524 (3)	
O1	0.3720 (3)	0.6242 (2)	−0.2597 (4)	0.0925 (9)	
O2	0.5564 (3)	0.6408 (2)	−0.1749 (5)	0.1230 (13)	
O3	0.14150 (17)	0.70007 (17)	0.3446 (2)	0.0596 (6)	
N1	0.4525 (3)	0.6311 (2)	−0.1531 (5)	0.0767 (9)	
N2	0.06689 (18)	0.63530 (16)	0.1098 (3)	0.0406 (5)	
H2	0.081 (2)	0.5858 (13)	0.052 (2)	0.033 (7)*	
N3	−0.08644 (18)	0.74365 (16)	0.1437 (3)	0.0438 (6)	
C1	0.3068 (2)	0.64030 (18)	0.0326 (3)	0.0441 (6)	
H1	0.2473	0.6492	−0.0509	0.053*	
C2	0.4242 (3)	0.6275 (2)	0.0067 (4)	0.0548 (8)	
C3	0.5143 (3)	0.6127 (2)	0.1286 (6)	0.0714 (11)	
H3	0.5926	0.6047	0.1090	0.086*	
C4	0.4858 (3)	0.6101 (3)	0.2790 (5)	0.0748 (11)	
H4	0.5450	0.5984	0.3621	0.090*	
C5	0.3705 (3)	0.6245 (2)	0.3079 (4)	0.0590 (8)	
H5	0.3525	0.6242	0.4105	0.071*	
C6	0.2803 (2)	0.63954 (18)	0.1845 (3)	0.0424 (6)	
C7	0.1583 (2)	0.6614 (2)	0.2225 (3)	0.0425 (6)	
C8	−0.0541 (2)	0.6519 (2)	0.1237 (3)	0.0395 (6)	
C9	−0.0126 (3)	0.8336 (2)	0.1380 (4)	0.0558 (8)	
H9A	0.0442	0.8261	0.0632	0.067*	
H9B	0.0300	0.8485	0.2403	0.067*	
C10	−0.1021 (3)	0.9124 (2)	0.0867 (4)	0.0656 (9)	
H10A	−0.1185	0.9147	−0.0267	0.079*	
H10B	−0.0738	0.9766	0.1249	0.079*	
C11	−0.2110 (3)	0.8824 (2)	0.1583 (5)	0.0689 (9)	
H11A	−0.2822	0.9090	0.0986	0.083*	
H11B	−0.2066	0.9057	0.2656	0.083*	
C12	−0.2117 (2)	0.7707 (2)	0.1528 (4)	0.0538 (8)	
H12A	−0.2389	0.7434	0.2464	0.065*	
H12B	−0.2629	0.7470	0.0616	0.065*	

### Atomic displacement parameters (Å^2^)

**Table:**

	*U*^11^	*U*^22^	*U*^33^	*U*^12^	*U*^13^	*U*^23^
S1	0.0347 (4)	0.0467 (4)	0.0771 (5)	−0.0080 (3)	0.0118 (3)	−0.0127 (4)
O1	0.106 (2)	0.100 (2)	0.0814 (19)	0.0194 (18)	0.0502 (17)	0.0123 (16)
O2	0.0760 (19)	0.122 (3)	0.189 (3)	0.0111 (17)	0.087 (2)	0.025 (2)
O3	0.0555 (13)	0.0768 (15)	0.0467 (12)	−0.0099 (10)	0.0069 (9)	−0.0160 (11)
N1	0.071 (2)	0.0548 (18)	0.114 (3)	0.0126 (15)	0.049 (2)	0.0172 (18)
N2	0.0311 (11)	0.0445 (13)	0.0471 (13)	−0.0040 (9)	0.0085 (9)	−0.0134 (10)
N3	0.0360 (12)	0.0451 (14)	0.0528 (14)	−0.0040 (9)	0.0145 (10)	−0.0083 (10)
C1	0.0323 (14)	0.0362 (15)	0.0641 (18)	−0.0005 (10)	0.0063 (12)	0.0047 (12)
C2	0.0433 (16)	0.0350 (15)	0.089 (2)	0.0002 (12)	0.0211 (16)	0.0048 (14)
C3	0.0308 (16)	0.0484 (19)	0.133 (4)	−0.0002 (13)	0.0038 (19)	−0.002 (2)
C4	0.0444 (19)	0.064 (2)	0.107 (3)	0.0044 (15)	−0.024 (2)	−0.006 (2)
C5	0.0539 (19)	0.0505 (18)	0.068 (2)	−0.0036 (14)	−0.0102 (15)	−0.0012 (15)
C6	0.0342 (14)	0.0356 (14)	0.0564 (17)	−0.0062 (10)	0.0016 (12)	−0.0010 (12)
C7	0.0398 (15)	0.0446 (15)	0.0429 (15)	−0.0078 (11)	0.0043 (12)	−0.0012 (12)
C8	0.0352 (13)	0.0474 (16)	0.0371 (13)	−0.0036 (11)	0.0091 (11)	−0.0051 (11)
C9	0.0541 (17)	0.0458 (17)	0.072 (2)	−0.0091 (14)	0.0265 (15)	−0.0099 (14)
C10	0.073 (2)	0.0493 (19)	0.077 (2)	−0.0027 (16)	0.0178 (18)	−0.0002 (16)
C11	0.065 (2)	0.056 (2)	0.089 (2)	0.0099 (16)	0.0211 (19)	−0.0054 (17)
C12	0.0409 (16)	0.0567 (19)	0.0661 (19)	0.0028 (13)	0.0155 (14)	−0.0051 (14)

### Geometric parameters (Å, º)

**Table:**

S1—C8	1.678 (3)	C4—C5	1.374 (5)
O1—N1	1.213 (4)	C4—H4	0.9300
O2—N1	1.222 (4)	C5—C6	1.393 (4)
O3—C7	1.209 (3)	C5—H5	0.9300
N1—C2	1.450 (5)	C6—C7	1.489 (4)
N2—C7	1.374 (3)	C9—C10	1.499 (4)
N2—C8	1.409 (3)	C9—H9A	0.9700
N2—H2	0.863 (10)	C9—H9B	0.9700
N3—C8	1.313 (3)	C10—C11	1.503 (5)
N3—C12	1.477 (3)	C10—H10A	0.9700
N3—C9	1.482 (3)	C10—H10B	0.9700
C1—C6	1.377 (4)	C11—C12	1.513 (4)
C1—C2	1.386 (4)	C11—H11A	0.9700
C1—H1	0.9300	C11—H11B	0.9700
C2—C3	1.384 (5)	C12—H12A	0.9700
C3—C4	1.372 (5)	C12—H12B	0.9700
C3—H3	0.9300		
			
O1—N1—O2	122.7 (4)	N2—C7—C6	115.7 (2)
O1—N1—C2	118.6 (3)	N3—C8—N2	116.9 (2)
O2—N1—C2	118.7 (4)	N3—C8—S1	123.67 (19)
C7—N2—C8	123.7 (2)	N2—C8—S1	119.4 (2)
C7—N2—H2	115.4 (16)	N3—C9—C10	103.3 (2)
C8—N2—H2	115.3 (16)	N3—C9—H9A	111.1
C8—N3—C12	121.9 (2)	C10—C9—H9A	111.1
C8—N3—C9	127.3 (2)	N3—C9—H9B	111.1
C12—N3—C9	110.3 (2)	C10—C9—H9B	111.1
C6—C1—C2	118.6 (3)	H9A—C9—H9B	109.1
C6—C1—H1	120.7	C9—C10—C11	104.3 (3)
C2—C1—H1	120.7	C9—C10—H10A	110.9
C3—C2—C1	121.9 (3)	C11—C10—H10A	110.9
C3—C2—N1	119.5 (3)	C9—C10—H10B	110.9
C1—C2—N1	118.6 (3)	C11—C10—H10B	110.9
C4—C3—C2	118.7 (3)	H10A—C10—H10B	108.9
C4—C3—H3	120.7	C10—C11—C12	105.0 (2)
C2—C3—H3	120.7	C10—C11—H11A	110.8
C3—C4—C5	120.5 (3)	C12—C11—H11A	110.8
C3—C4—H4	119.8	C10—C11—H11B	110.8
C5—C4—H4	119.8	C12—C11—H11B	110.8
C4—C5—C6	120.5 (3)	H11A—C11—H11B	108.8
C4—C5—H5	119.8	N3—C12—C11	104.4 (2)
C6—C5—H5	119.8	N3—C12—H12A	110.9
C1—C6—C5	119.8 (3)	C11—C12—H12A	110.9
C1—C6—C7	121.7 (2)	N3—C12—H12B	110.9
C5—C6—C7	118.4 (3)	C11—C12—H12B	110.9
O3—C7—N2	122.5 (2)	H12A—C12—H12B	108.9
O3—C7—C6	121.8 (2)		
			
C6—C1—C2—C3	0.9 (4)	C5—C6—C7—O3	26.7 (4)
C6—C1—C2—N1	−178.3 (2)	C1—C6—C7—N2	30.6 (4)
O1—N1—C2—C3	165.5 (3)	C5—C6—C7—N2	−153.7 (2)
O2—N1—C2—C3	−14.5 (4)	C12—N3—C8—N2	177.3 (2)
O1—N1—C2—C1	−15.3 (4)	C9—N3—C8—N2	6.5 (4)
O2—N1—C2—C1	164.7 (3)	C12—N3—C8—S1	−1.5 (4)
C1—C2—C3—C4	0.5 (5)	C9—N3—C8—S1	−172.2 (2)
N1—C2—C3—C4	179.7 (3)	C7—N2—C8—N3	58.4 (4)
C2—C3—C4—C5	−1.7 (5)	C7—N2—C8—S1	−122.9 (2)
C3—C4—C5—C6	1.6 (5)	C8—N3—C9—C10	152.1 (3)
C2—C1—C6—C5	−1.0 (4)	C12—N3—C9—C10	−19.5 (3)
C2—C1—C6—C7	174.7 (2)	N3—C9—C10—C11	33.2 (3)
C4—C5—C6—C1	−0.2 (4)	C9—C10—C11—C12	−35.2 (4)
C4—C5—C6—C7	−176.1 (3)	C8—N3—C12—C11	−174.1 (3)
C8—N2—C7—O3	0.9 (4)	C9—N3—C12—C11	−2.0 (3)
C8—N2—C7—C6	−178.8 (2)	C10—C11—C12—N3	22.8 (4)
C1—C6—C7—O3	−149.1 (3)		

### Hydrogen-bond geometry (Å, º)

**Table:**

*D*—H···*A*	*D*—H	H···*A*	*D*···*A*	*D*—H···*A*
N2—H2···S1^i^	0.86 (2)	2.55 (2)	3.406 (3)	173
C1—H1···O3^ii^	0.93	2.48	3.169 (3)	131
C9—H9*A*···O3^ii^	0.97	2.32	3.274 (4)	167

Symmetry codes: (i) −*x*, −*y*+1, −*z*; (ii) *x*, −*y*+3/2, *z*−1/2.
